# Medical News

**Published:** 1852-11

**Authors:** 


### MEDICAL NEWS.

At a meeting of the Students of the Medical Department of the University of New York, held Oct. 18th, 1852, the following preamble and resolutions were unanimously adopted:

*Whereas,* The successful practice of the "Healing Art" imperatively demands a knowledge of the functions of the various organs of the animal economy, which branch of Medical knowledge has been too much neglected, but is now justly engaging the attention of, and becoming properly appreciated by the Profession; and, *Whereas,* Dr. BROWN SEQUARD, of Paris, in his experiments and investigations for the advancement of Physiological science, has arrived at conclusions, and rendered truths demonstrable, which have been heretofore unknown or conjectural; Therefore,

*Resolved,* That Dr. SEQUARD is entitled to the commendation of the Medical Profession, and merits their approbation; and further

*Resolved,* That we hereby tender him our sincere and heartfelt thanks, as an humble testimonial of our appreciation of his instructive and interesting series of Lectures before the class.

*Resolved,* That while our thanks are eminently due to Dr. SEQUARD, they are no less due to the Faculty of the University for his introduction to the class, as well as for their untiring efforts in sustaining in an able and superior manner the past summer's course of lectures.

*Resolved,* That we hereby tender them our most hearty thanks.

*Resolved,* That a copy of these resolutions be presented to Dr. SEQUARD and the Faculty of the University, and published in the Herald and Tribune. Signed in behalf of the class.

P. T. TUNISON, Chairman.

H. HALSEY TICHENOR, Secretary.

TRIBUTE TO DR. HOOKER.—At a meeting of the Norwich Medical Association, August 12th, 1852, a committee was appointed to draft resolutions in regard to the anticipated removal of Dr. WORTHINGTON HOOKER to New Haven. The following, proposed by the committee, were unanimously adopted, and ordered to be published in such journals as the committee should think proper.

*Whereas,* The Members of the Norwich Medical Association, desiring to avail themselves of the opportunity for expressing their regard for Dr. Hooker on the occasion of his retiring from the practice of his profession in this place, and feeling warmly attached to him personally, for his kind professional intercourse and the uniform courtsey and patience which have distinguished him in the discharge of his duties, and having a high esteem and regard for him as a talented and accomplished physician,—therefore,

*Resolved,* That we sincerely regret the separation from us of one who has set so eminent an example of true professional honor, and who has ever so scrupulously regarded the rights and feelings of his medical brethren.

*Resolved,* That such an example puts to shame too many medical practitioners of the present day, who seem to be governed by an overbearing spirit, or by a crafty, insinuating disposition, wounding at every step the fair fame of those whom they should treat as brethren.

*Resolved,* That in his work entitled "PHYSICIAN AND PATIENT," Dr. Hooker exhibited rare qualities that have justly secured to him the esteem and confidence of the great body of the profession throughout the country; and that the prominent position which he now holds among medical men, resulting from these qualities, indicates for him a career of great and extensive usefulness in the new relation to which he has been called.

*Resolved,* That in Dr. Hooker's untiring zeal and industry, in his careful habits of investigation, in his sound and wise discrimination in the application of medical principles, and in the operations of his clear and vigorous mind, enlightened and instructed by the various learning of his profession, we see the true causes of the eminence to which he has attained.

*Resolved,* That we congratulate the Faculty of Yale College, and the Medical Profession in the city of New Haven, upon their good fortune in securing for the Chair of the Theory and Practice of Medicine a gentleman so fully possessing the requisite qualities for filling that position.

HORACE THURSTON,
Norwich, Sept. 17th, 1852. *Secretary of the Association.*

APPOINTMENT.—The Board of Trustees of the New Hampshire State Lunatic Asylum have elected JOHN E. TYLER, M. D., Superintendent of that Institution.

EMIGRANTS' HOSPITAL, WARD'S ISLAND, N. Y.—Dr. Thomas Addis Emmet has been appointed one of the "Visiting Physicians" to this Hospital. Dr. Emmet has won the confidence of the Board of Managers by his constant and unremitting devotion to his duties, as resident physician during the past two years, and has been rewarded by this high honor, rarely bestowed on one so young in years and the profession.

NEW MEDICAL SCHOOL.—A new Medical School has been established at Cincinnati, styled the "*Miami Medical College of Cincinnati.*" The Professors are the venerable and distinguished Dr. Mussey, and Drs. Judkins, Avery, Davis, White (late of Philadelphia,) Mendenhall, Murphy, Comegys, and Locke, Jr.

PROFESSORIAL APPOINTMENTS.—Dr. J. LAWRENCE SMITH, of the University of Louisiana, has been elected to succeed Dr. R. E. ROGERS, in the Chair of Chemistry in the University of Virginia. This appointment is highly satisfactory to the friends of this excellent Institution.

Drs. PALMER and FLINT, of the University of Buffalo, have succeeded Drs. Cobb and Drake in the University of Louisville, the latter gentlemen having accepted Chairs in the Medical College of Ohio.

Dr. E. M. MOORE, of Woodstock, Vt., takes the Chair of Anatomy in the University of Buffalo, vacated by Dr. Palmer.

TRANSYLVANIA MEDICAL JOURNAL.—Dr. L. J. FRAZEE has assumed the editorial duties of this Journal.

CHOLERA.—The latest intelligence from Prussia is rather satisfactory as regards the progress of cholera, for although it has spread through the Polish territories, not only has its virulence abated, but the number of cases has also diminished. At Berlin three hospitals have been opened, and at Posen, from the 20th of July to the 20th of September, there were 2491 cases and 1235 deaths. All the public schools have been closed; and at Konisberg, M. Von Treschowitz and M. Waldow connected with the legislature, have died. At Posen, the wife of M. Von Pattkamaner, the President, also died of cholera.

BARON HUMBOLDT attained his 83rd birth-day on the 14th of Sept. This "Nestor of Science" is in excellent health, and is engaged daily for hours on a fourth volume of "Cosmos."

MEDICAL FEES IN SPAIN.—The fee of a Spanish physician at present is said to be twopence from a tradesman, tenpence from a man of rank, and nothing from the poor. In France, the fee from a tradesman is from three to five francs; from a man of rank and wealth the amount varies, large sums being occasionally given.

M. WURTZ, Professor at the Ecole de Médécine, has succeeded in extracting from the oil of potatoes a new alcohol, which he calls *Alcoöl entylique*. It is obtained by repeated distillations. Its composition is $C_8\ H_{10}\ O_2$.

M. DIZE, a distinguished chemist, Member of the Académie de Médecine, and of various learned societies of France, died lately, aged 88. When yet a young chemist, he established the manufacture of artificial soda, which has become to France the source of a revenue of a million of francs. On the breaking out of the first Revolution, he organized at the Ecole Militaire the central pharmacy of the armies of the Republic. He also invented new processes for refining gold and silver, and did various services for public departments of the State.—*London Med. Times.*

The Autobiography of Berzelius.—After the death of this great Swedish chemist, it was found that he had left behind him a copious and highly interesting biography, for the publication of which we have long and anxiously waited. It appears, however, that the autobiography contains so many personal allusions, and so many unsparing criticisms of living or recently deceased scientific men, that the executors are not only afraid of publishing, but are actually prevented from doing so by the laws of Sweden, which forbid the printing of works dealing in personalities, until several years after the death of their authors. From the known dislike of Berzelius to Sir Humphry Davy, and to the recently deceased Dr. Thomas Thomson, it is supposed that these eminent chemists figure largely in the suppressed work. We feel sure that the surviving relatives of these distinguished men would prefer the opportunity of answering such criticism themselves, rather than leave the task to a future generation. We trust that the obstacles which prevent the appearance of this—as we are told—remarkable work, may be overcome, and that speedily.—*London Lancet.*

